# Indirect exposure to insect growth disruptors affects honey bee (*Apis mellifera*) reproductive behaviors and ovarian protein expression

**DOI:** 10.1371/journal.pone.0292176

**Published:** 2023-10-02

**Authors:** Julia D. Fine, Leonard J. Foster, Alison McAfee

**Affiliations:** 1 Invasive Species and Pollinator Health Research Unit, USDA-ARS, Davis, CA, United States of America; 2 Department of Biochemistry and Molecular Biology, Michael Smith Laboratories, University of British Columbia, Vancouver, BC, Canada; 3 Department of Applied Ecology, North Carolina State University, Raleigh, NC, United States of America; University of Carthage, TUNISIA

## Abstract

Pesticide exposure and queen loss are considered to be major causes of honey bee colony mortality, yet little is known regarding the effects of regularly encountered agrochemicals on honey bee reproduction. Here, we present the results of a two-generational study using specialized cages to expose queens to commonly used insect growth disrupting pesticides (IGDs) via their retinue of worker bees. Under IGD exposure, we tracked queen performance and worker responses to queens, then the performance of the exposed queens’ offspring was assessed to identify patterns that may contribute to the long-term health and stability of a social insect colony. The positive control, novaluron, resulted in deformed larvae hatching from eggs laid by exposed queens, and methoxyfenozide, diflubenzuron, and novaluron caused a slight decrease in daily egg laying rates, but this was not reflected in the total egg production over the course of the experiment. Curiously, eggs laid by queens exposed to pyriproxyfen exhibited increased hatching rates, and those larvae developed into worker progeny with increased responsiveness to their queens. Additionally, pyriproxyfen and novaluron exposure affected the queen ovarian protein expression, with the overwhelming majority of differentially expressed proteins coming from the pyriproxyfen exposure. We discuss these results and the potential implications for honey bee reproduction and colony health.

## Introduction

As agricultural pollinators, honey bees are critical to ensuring that global demands for food production are satisfied [1–4]. However, as a consequence of their use as pollinators, honey bees are often exposed to agrochemicals used to control pests in their foraging environment [5–7]. Exposure to pesticides, including those used in agricultural crops, has been identified as a major contributor to honey bee colony loss [8–10]. Much is known regarding the lethality of high doses of agrochemicals (especially neonicotinoids) on adult worker bees [11–14], but relatively less is understood regarding their sublethal effects [15], perhaps because there are many more endpoints to be considered or because sublethal assessments are not always required to register a pesticide [16]. Even less well understood are the effects of agrochemicals on honey bee queens and colony reproduction [17–19].

Within a colony, reproduction is heavily dependent upon the activity and health of a single, mated queen who is solely responsible for laying fertilized eggs, which hatch and develop into worker bees that make up the bulk of the colony [20]. However, both the queen and the larvae she produces rely upon the coordinated efforts of worker bees for provisioning and care, making the activities inside the nest the result of complex and codependent relationships between the queen, workers and larvae [21, 22]. These relationships are governed by numerous inputs including chemical [23–26], nutritional [27–29], and seasonal signals [30, 31]. However, these signals and the ways in which bees to respond to them can change depending on biological factors such as the health [32], reproductive potential [33, 34], nutritional status [35], and age of honey bees [36]. Given the demonstrated effects of some agrochemicals on aspects of honey bee reproduction [37–41], it is important to consider how pesticides can affect reproductive behaviors such as queen retinue performance and oviposition, as well as how maternal exposure scenarios may affect the health, physiology, and behavior of her offspring.

Agrochemicals are known to have a range of sublethal effects on arthropods, including altered behavior [42–45], changes in feeding patterns [46–49], attractancy and repellency [6, 50, 51], and decreased fecundity [47, 52, 53]. Over the past decade, negative effects of pesticides encountered in worker diet, wax, or generally in the honey bee foraging environment have been identified, impacting metrics like colony expansion and queen supersedure [37–39], decreased sperm viability and reproductive success [40], decreased oviposition [41, 49], queen pheromone production and worker attraction [54, 55], and larval provisioning [56]. Broadly, these studies suggest that in addition to causing toxic effects in adult bees, pesticides affect other members of the colony such as the queen and young larvae, which were previously thought to be somewhat insulated against the most harmful effects of pesticide poisoning by the worker bees that provision and care for them [57]. Because the continued functioning of a honey bee colony depends on the coordinated behaviors of thousands of individuals [21], changes in these behavioral patterns can potentially be devastating to the long-term health of the colony [22, 58].

In this work, we examined the reproductive toxicity to honey bees of three different insect growth disruptor pesticides (IGDs) including the chitin synthesis inhibitor diflubenzuron and the insect hormone mimics pyriproxyfen and methoxyfenozide, all administered at 1 ppm through the workers’ diet, thus exposing queens indirectly in a manner similar to what might occur in the field. Previous work has shown IGDs administered at ten times this concentration can negatively affect egg hatching rates [59], and this lower dose was selected to assess the potential for effects in surviving embryos. This dose is higher than what has been reported for these compounds in samples taken from inside colonies such as wax or stored food [60–62], but field collected pollen can contain residues of IGDs in this range [63]. These IGDs were selected due to their frequent use in almond orchards to control a variety of immature pests during or near the time of bloom [64], when over one million honey bee colonies are used for pollination [65]. Additionally, the chitin synthesis inhibitor novaluron was administered as a positive control at 20 ppm, a concentration known to disrupt brood production in nucleus colonies [63].

Using specialized queen monitoring cages (QMCs), which are similar to microcolonies containing a mated queen and a small compliment of young workers [49, 66], we tracked and monitored oviposition and queen and worker interactions, then used quantitative proteomics to detect changes in the queens’ ovaries. Additionally, we used unexposed colonies to rear larvae from eggs laid by exposed queens, then examined the transovarial effects of the IGDs by monitoring the behavior of the resultant adults when interacting with a novel, unexposed queen. Although oviposition rates of queens are generally lower for queens in QMCs compared to full-sized colonies [67], previous work has shown that QMCs can be used to mimic real-world responses to stressors [49, 66, 68] and to study complex biological phenomena [69]. The results presented here explore not only the effects of IGDs on queens and workers but on the next generation of workers as well, generating insight into the potential long-term effects of IGD exposure on honey bees.

## Methods

This study was conducted in two parts: Round 1 and 2. In Round 1, we examined effects of IGD exposure through worker diet on queen performance and worker retinue behavior, and in Round 2, we examined vertical effects of indirect queen exposure on her progeny. In Round 1, queens in QMCs were exposed to IGDs indirectly via worker attendants feeding on IGD-laced diets. Eggs laid by these queens were then fostered and reared to adulthood in untreated colonies. In Round 2, these fostered workers were introduced to new QMCs with unrelated queens.

### Round 1: Putative queen exposure via workers

#### Honey bees

**Fig 1:**
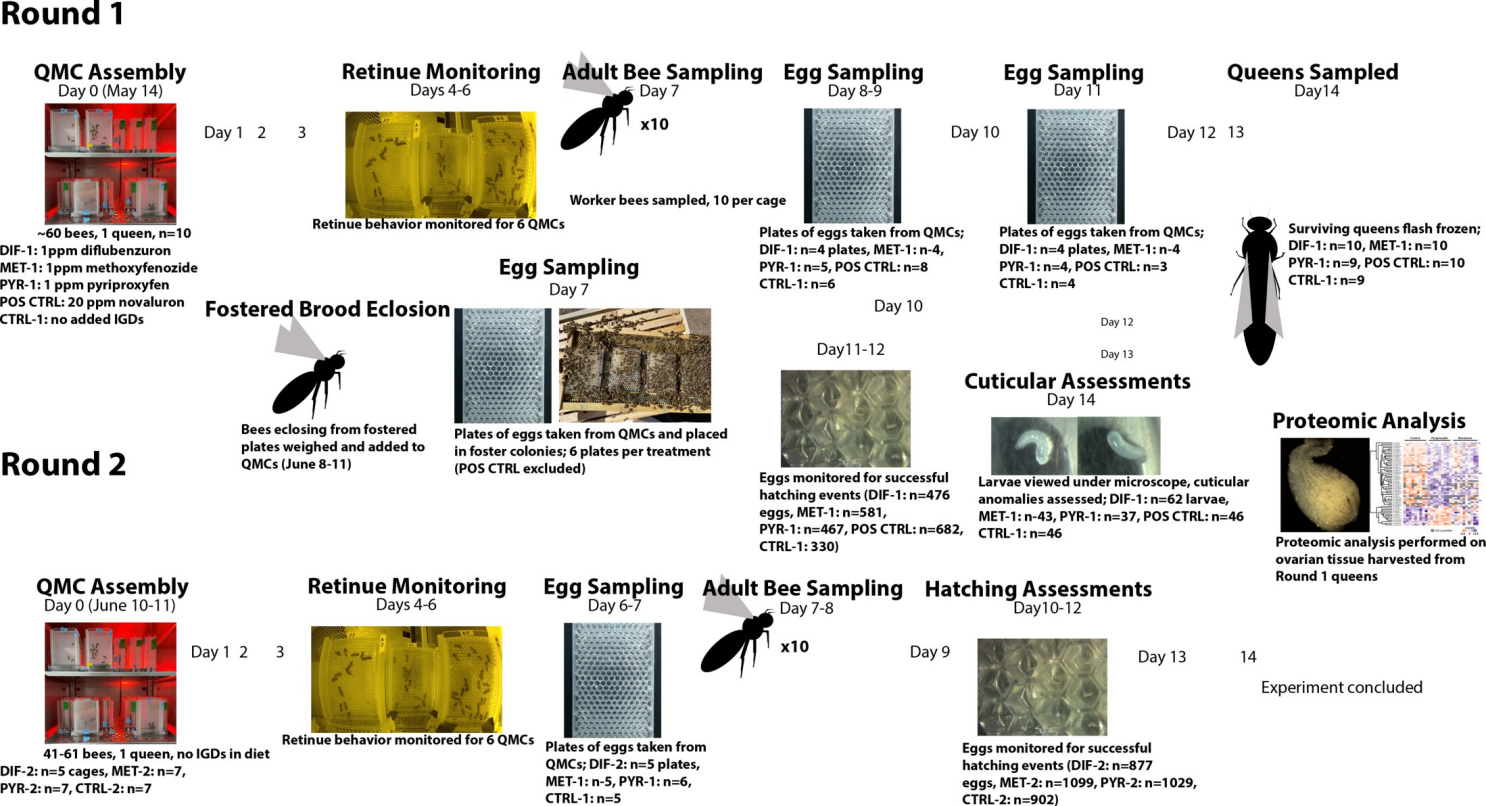
Graphical representation of experimental timeline.

Honey bee colonies of primarily Italian lineage were kept at the Harry H. Laidlaw Jr. Honey Bee Research Facility at the University of California Davis. On April 22, 2021, five queens were caged on frames of empty wax comb in their respective colonies and released two days later, after laying eggs onto the comb. On May 12, the same frames, now containing capped worker brood, were removed from the colonies and placed in an incubator set at 34.5°C until they eclosed. On May 14, newly eclosed bees were brushed from the frames into the same container, allowed to mix for approximately 30 minutes, and placed in QMCs. See Fig 1 for a comprehensive graphical depiction of our experimental design and timeline.

#### QMC assembly

QMC assembly, maintenance and monitoring methods were adapted from Fine et al. [49]. On May 14 (day 0), 6 g of newly eclosed bees (approximately 60 bees [70, 71]) and a single mated honey bee queen acquired within 48 h from a queen producer (Northern California) were introduced to each QMC. Mated queens were anesthetized with CO_2_ for approximately 30 seconds, weighed, and paint marked with an oil-based paint pen (Sharpie, Newell Brands, Atlanta, GA) before being added to the cages. Each QMC was given two feeders containing 50% (wt/wt) aqueous sucrose solution, one feeder containing water, and one feeder containing MegaBee© pollen supplement prepared by mixing 50% sucrose solution with the powdered supplement at a 1:1 ratio by weight (MegaBee LLC, San Diego, CA). Each cage contained one new egg laying plate (designed to mimic the dimensions of natural wax comb but composed of polystyrene with 264 wells [66]) when the experiment began, allowing queens to lay up to 264 eggs during a monitoring period. After day 6, if queens laid more than 100 eggs during a daily monitoring period, they were given an additional plate to facilitate increases in oviposition. Each treatment group consisted of 10 QMCs maintained in incubators for 14 days. One queen death occurred during the monitoring period in the control treatment group. The cage was removed from the experiment and the data were thus censored.

#### Incubators

QMCs and were primarily maintained in a Heratherm^TM^ (ThermoFisher Scientific, Waltham, MA) incubator maintained at 34 ± 0.5°C. To minimize disruptions due to a novel stimulus while imaging, the cages were kept under constant red light with a 9 W red bulb (Amazon Basics, Seattle, WA) suspended from a narrow inlet at the back of the incubator near the top (incubator 1). Relative humidity (RH) was maintained at 75 ± 10% [49] using a saturated sodium chloride aqueous solution placed in the bottom of the incubator. For retinue monitoring, six QMCs from each treatment were moved to a second Heratherm incubator maintained at identical conditions and arranged across four shelves (incubator 2). Each shelf was illuminated by a 9 W bulb as described above. To reduce glare, black construction paper was strategically hung along the inner glass door of the incubator. For more details on retinue monitoring see the section “Camera Monitoring–Retinue Behavior” below.

#### Treatments

Treatments were chronically administered via sucrose solution and pollen supplement diet. Treatment groups consisted of 10 cages exposed to either diflubenzuron (DIF-1, Sigma-Aldritch, St. Louis, MO, 98% purity), methoxyfenozide (MET-1, Sigma-Aldritch, St. Louis, MO, 98% purity), or pyriproxyfen (PYR-1, Sigma-Aldritch, St. Louis, MO, 98% purity) each at 1 ppm (wt/wt), or novaluron (POS-CTRL, Sigma-Aldritch, St. Louis, MO, 98% purity) at 20 ppm novaluron (administered as a positive control). For diflubenzuron, methoxyfenozide, and pyriproxyfen-laced diets, 0.01 g of technical grade chemical was first dissolved in 10 mL of 1:1 acetone and methanol solution (hereon referred to as ‘solvent’) due to the limited solubility of methoxyfenozide in pure acetone. For the POS-CTRL-treated sucrose solution, 0.2 g of technical grade chemical were dissolved in 10 mL of solvent. Next, 100 μl of each stock solution was added to 100 g of sucrose solution to produce sucrose diets with final concentrations of 1 ppm (wt/wt) DIF, MET, and PYR and 20 ppm POS-CTRL. For pollen supplement diets, 10 μl of stock solution were added to 5 g of sucrose solution, mixed thoroughly, added to 5 g of powdered supplement and again mixed thoroughly. An equivalent volume of solvent was added to sucrose solution and pollen supplement for the control treatment group (CTRL). Acetone (up to 1%) is the preferred solvent for standardized oral toxicity tests on honey bees [72], and while there is little information available regarding the effects of methanol on honey bees, it appears to be nontoxic to honey bee larvae [73], which have a reduced capacity for the activity of certain detoxification enzymes relative to adults [74], at a concentration of 1%. Here, we use a concentration of 0.1% in diet and did not use an additional control without solvent. All feeders were refilled daily as needed and replaced every two days for the duration of the experiment.

#### Egg production, mortality and pollen supplement consumption

Egg production, worker mortality and pollen supplement consumption were quantified according to methods described in Fine et al. [49]. Briefly, QMCs were removed from the incubator in the morning and eggs present in each egg laying plate were counted and removed daily. Unless the eggs were sampled to assess viability or rear to adult eclosion, egg removal was performed destructively. At this time, the number of dead bees in each QMC were counted and removed through feeder holes. Pollen supplement consumption was measured every other day by weighing feeder tubes before placing them into QMCs and recording the difference after they were removed two days later. QMCs were never outside of the incubator for more than 15 minutes while performing all quantitative assessments or sampling.

#### Camera monitoring–retinue behavior

**Fig 2:**
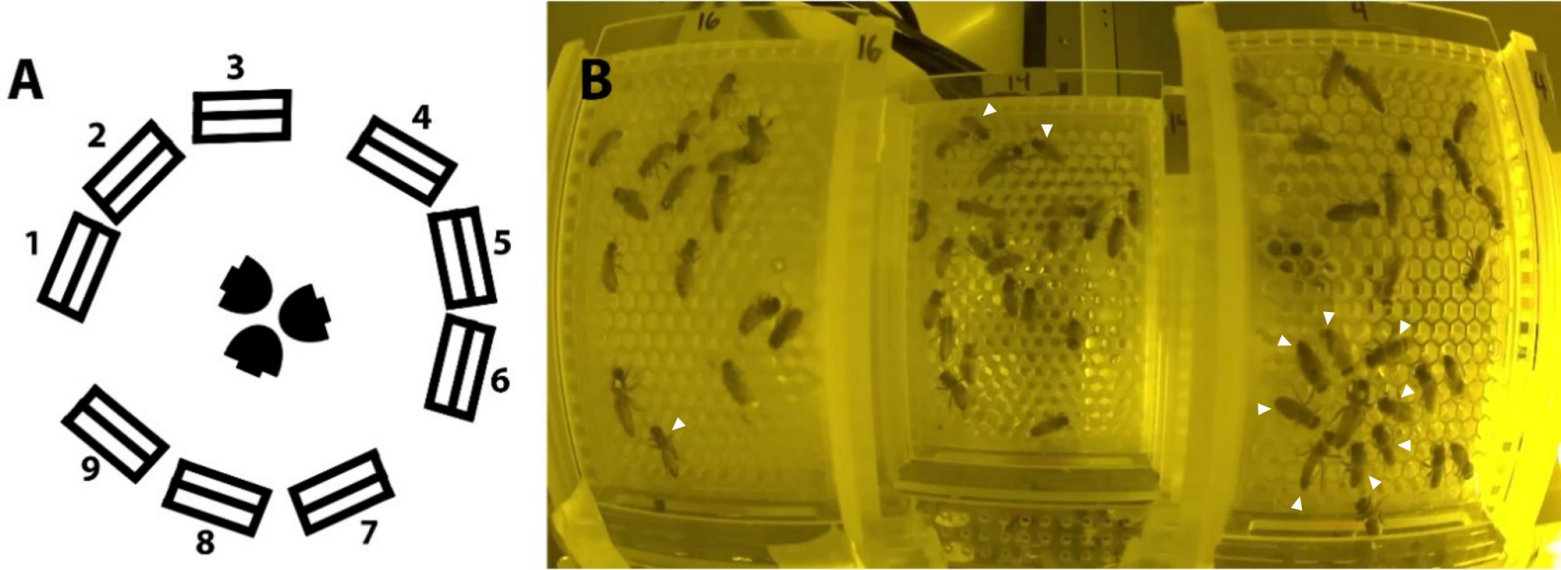
The general arrangement of QMCs in incubator 2 for monitoring retinue behavior. A. The spacing of the maximum number of QMCs (spaces 1–9) and cameras (center) across a single shelf. B. An image taken during retinue monitoring under red light depicting marked queens and unmarked workers. “Interacting” workers are marked with white arrows.

On day 4 of the experiment, six QMCs per treatment group were transferred to incubator 2 following daily egg production checks and arranged as shown in Fig 2A for retinue monitoring. The treatments were dispersed evenly across the shelves and arranged so that the clear panel over the egg laying chambers was visible to one of up to three Arlo model VMS440P cameras (Arlo Technologies, San Jose, CA) placed in the center of each shelf. See Fig 2 and S1 Table for more details on the arrangement of cages inside the monitoring incubator.

Following a 30 min adjustment period, cameras were accessed remotely and images of the workers and queens inside QMCs were taken, with each camera capturing a maximum of three QMCs in each photo (Fig 2B). Images were taken every 30 s for five min before moving on to the next set of QMCs until all QMCs had been imaged for five minutes. This cycle was repeated three times for a total of 30 images per QMC, and QMCs were then moved back to incubator 1 until the next monitoring period. This procedure was repeated on days 5 and 6.

The retinue responses of worker bees to queens in each image was scored by counting the number of workers licking, grooming, antennating, or otherwise interacting with or facing the queen within an approximate distance of one cell. These observations were recorded by observers blinded to treatment.

#### Embryo viability and queen and worker sampling

On day 7, 10 workers were sampled from each QMC for future work. On days 8 and 9, plates of eggs were sampled from four to eight QMCs per treatment (4 queens DIF-1, 4 MET-1, 5 PYR-1, 8 POS-CTRL, and 6 CTRL-1) depending on the number of eggs available, and hatching rates were assessed according to methods in Fine 2020 [59]. Briefly, egg laying plates were covered with a PCR plate seal and a universal plate lid after removing them from QMCs and replacing them with new, empty plates. The plates containing eggs were placed in a chamber maintained at 95% RH with a saturated potassium sulfate solution inside incubator 1. Approximately 72 h later, the egg laying plates were removed to assess hatching rates. Each egg laying plate was kept outside of the incubator for no longer than 25 min for these assessments before covering the plates and returning them to incubator 1. The following day, hatching rates were assessed again. In total, between 330–682 eggs were assessed per treatment (n = 476 eggs DIF-1, 581 MET-1, 467 PYR-1, 682 POS-CTRL, 330 CTRL-1, S1 Data).

**Fig 3:**
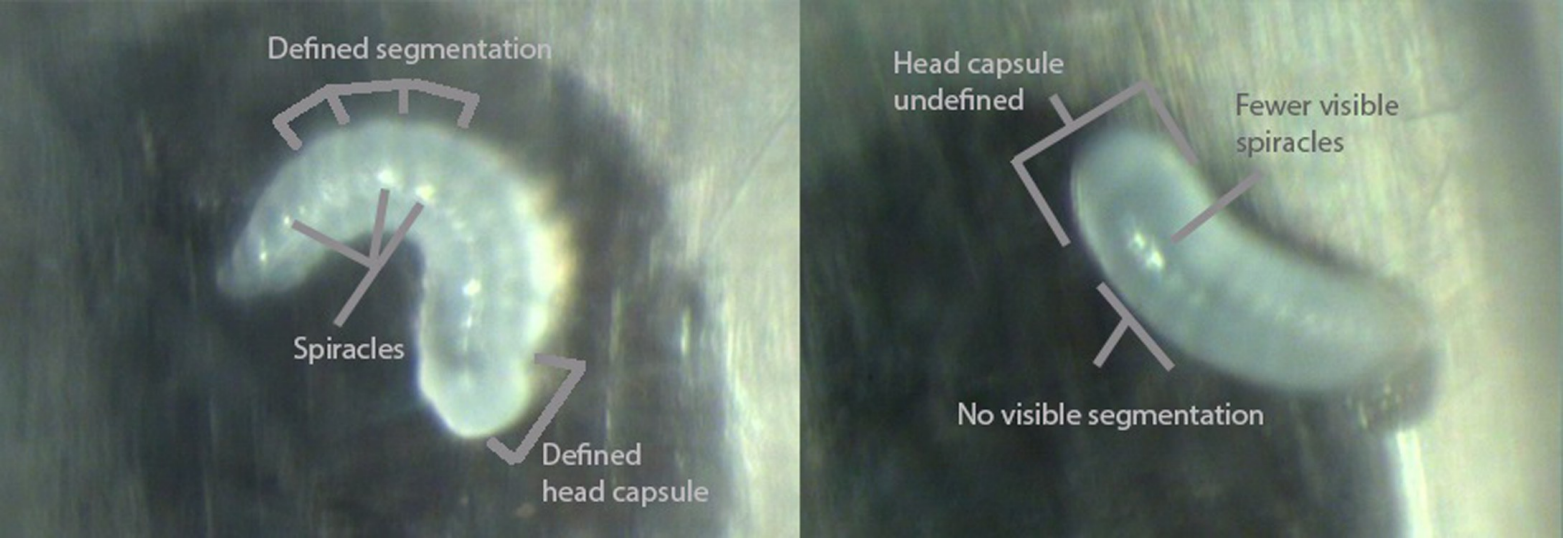
Images of larvae hatched from eggs laid by queens exposed to IGDs in Round 1. Left: Larva with apparently normal anatomy. Segmentation and anatomical features like spiracles and head capsule are discernable and larva is curled in a “c” shape. Right: Deformed larva. Smooth cuticle without the appearance of segmentation. Anatomical features like spiracles and head capsule are difficult to discern. Larvae has failed to curl into a “c” shape after hatching.

On day 11, three to four plates of eggs per treatment (4 plates DIF-1, 4 MET-1, 4 PYR-1, 3 POS-CTRL, and 4 CTRL-1) were sampled from QMCs to assess deformities of the cuticle in newly hatched larvae (see Fig 3 for a description of noted deformities). Egg laying plates were maintained as described above, and after approximately 72 h, newly hatched larvae on each plate were randomly selected for closer examination under a microscope. Depending on the number of hatched larvae available, 3–29 larvae per plate or 37–62 larvae per treatment were imaged (S1 Data). Images were taken of the larvae at 16x magnification using an Olympus SZX10 microscope and an EP50 camera (Olympus Corporation, Tokyo, Japan) and the presence of deformed cuticle or normal anatomy (Fig 3) was recorded by an observer blind to treatment groups. On day 14, queens were removed from QMCs, weighed, and flash frozen for proteomic analysis.

#### Proteomics sample preparation

All queens that were alive at the conclusion of Round 1 (except for 1 PYR-1 queen lost during sampling) were shipped on dry ice to the University of British Columbia, where ovaries were dissected from previously frozen queens (n = 10 queens DIF-1, 10 MET-1, 9 PYR-1, 10 POS-CTRL, and 9 CTRL-1) under a dissecting scope and weighed. Ovaries were then placed in tissue extraction tubes (2.0 mL DuraTube) containing 800 μl of guanidinium chloride extraction buffer (6 M guanidinium chloride, 100 mM Tris, pH 8.0) and four ceramic homogenization beads. Proteins were extracted, precipitated and solubilized as previously described [75], with some modifications. Briefly, after homogenization, the sample was clarified by centrifugation (16,000 *g*, 10 min) and 100 μl of the supernatant under the upper fatty layer was removed and mixed with 100 μl of ultrapure water ahead of acetone precipitation (800 μl of ice-cold, 100% acetone; -20°C, overnight). The precipitated protein was pelleted (5,000 *g*, 15 min) and washed three times with 500 μl of 80% acetone (ice-cold), ensuring the pellet was disrupted again with each wash. The rest of the extraction and digestion protocol was performed as previously described [75]. The protein was solubilized in urea buffer (6 M urea, 2 M thiourea, 100 mM Tris, pH 8.0), quantified using a Bradford assay, 25 μg of each sample was reduced using dithiothreitol, alkylated using iodoacetamide, and digested with lys-C and trypsin overnight. Peptides were desalted using the stop-and-go-extraction (STAGE) tip method [76] and quantified using a nanodrop (A280 nm).

Desalted peptides (300 ng suspended in buffer A: 0.1% formic acid, 2% acetonitrile in ultrapure water) were injected on a liquid chromatography system (Easy-nLC 1200; Thermo Fisher Scientific, Waltham, MA) coupled to an Impact II Q-TOF mass spectrometer (Bruker Corporation, Billerica, MA). Samples were injected in randomized order. The LC system used a CaptiveSpray ionization source (Bruker Daltonics) and an Aurora Series Gen2 (CSI) analytical column, (25cm x 75μm 1.6μm FSC C18, with Gen2 nanoZero and CSI fitting; Ion Opticks, Parkville, Victoria, Australia). The analytical column was heated to 50°C using a tape heater (SRMU020124, Omega.com, and in-house build microprocessor temperature controller). The separation gradient ran from 5–13% buffer B over 45 min, then to 35% buffer B from 45 to 90 min, then to 90% buffer B over 2 min, and held at this solvent for 13 min. The LC flow rate was 0.4 μL/min. Before each run, the analytical column was conditioned with 4 μL of buffer A.

The Impact II was run with oTOF Control v. 5.2 (Bruker). The LC and MS systems were controlled with HyStar 5.0 SR1 (5.0.37.0, Bruker). The Impact II was set to acquire in a data-dependent auto-MS/MS mode with inactive focus fragmenting the 20 most abundant ions (one at the time at 18 Hz rate) after each full-range scan from m/z 200 Th to m/z 2000 Th (at 5 Hz rate). The isolation window for MS/MS was 2 to 3 Th depending on parent ion mass to charge ratio and the collision energy ranged from 23 to 65 eV depending on ion mass and charge. Parent ions were then excluded from MS/MS for the next 0.3 min and reconsidered if their intensity increased more than five-fold. Singly charged ions were excluded and strict active exclusion was applied. The error of mass measurement was typically within 5 ppm and not allowed to exceed 10 ppm. The CaptiveSpray source was operated at 1900 V capillary voltage, 0.25 Bar pressure with methanol in the nanoBooster, 3 L/min drying gas, and 150°C drying temperature. More detailed instrument parameters can be found within the “microTOFQImpactAcquisition.method” file located within the .m folder of each sample’s publicly available data folder (hosted by MassIVE (https://massive.ucsd.edu/ProteoSAFe/static/massive.jsp), accession: MSV000089189).

#### Data processing

Mass spectrometry data were searched as previously described [77] using MaxQuant (v 1.6.1.0) [78] with default parameters, except that “match between runs” and “label-free quantification” were enabled. The data were searched against the honey bee reference proteome available on NCBI (based on the genome build HAv3.1) with all honey bee virus and *Nosema* proteins included in the fasta file (*A. mellifera*: 23,460; *N. apis*: 3,095; *N. ceranae*: 10,048; DWV: 138; SBV: 232; ABPV: 6; BQCV: 35; VDV: 3; KBV: 33; IAPV: 91), which is available with the raw data hosted on MassIVE (MSV000089189). All sequences were downloaded on Nov 18^th^, 2019. The proteinGroups.txt file was imported to Perseus (v 1.6.1.1) [79], where proteins only identified by site, potential contaminants, and reverse hits were removed, followed by proteins identified in fewer than three replicates of each group. This filtered the total protein groups from 2,816 down to 1,451 quantified protein groups, which is the set upon which we performed our statistical analysis.

### Round 2: Monitoring workers following putative maternal exposure

#### Larval fostering

On May 21, egg laying plates were removed from the six Round 1 QMCs that had the highest number of eggs in DIF-1, MET-1, PYR-1, and CTRL-1 treatment groups and replaced with new, empty plates. POS-CTRL was excluded as the larvae were almost all non-viable. The plates containing eggs were labeled and pressed into frames of wax comb with two on each side so that the eggs faced outward and each frame contained one plate from each treatment group. Each of the six frames were placed in separate foster colonies.

On June 3, 13 days after the eggs were laid into the egg laying plates, the frames were removed from the foster colonies and the plates, now containing capped brood, were placed in live insect containers (BioQuip, Rancho Dominguez, CA) measuring 17.78 x 11.43 x 12.70 cm. Each container housed two plates from the same treatment group with the capped cells facing inwards and maintained in incubator 1 until adult bees eclosed from the plates. See S2 Table for details including the QMC source, number of eggs on each plate, and the number of bees eclosing in each container.

#### QMC assembly

Bees eclosed in their containers from June 8 to 11, or 18–21 after eggs were laid, and the resulting adult workers were placed in QMCs according to maternal treatment with 1/10^th^ of a Tempqueen lure (INTKO Supply, Chilliwack, BC) on a safety pin suspended with fishing line from the top of the cage. These lures, which are impregnated with a synthetic blend of nine-component queen pheromone [80] equivalent to that of ten queens, were administered to newly eclosed bees in lieu of a mated queen because early exposure to queen pheromone has been shown to affect the behavioral development of young worker bees [23]. Upon eclosion, up to 10 bees per container per day were weighed, then added to QMCs. Eclosing bees were checked twice per day, once in the morning and once in the evening. Between 55 and 61 bees were added to each QMC, except one cage containing workers from eggs laid by queens exposed to diflubenzuron, which only contained 41 bees. Omitting this cage did not appreciably change the results of analyses of egg production and retinue response and it was therefore not censored from any datasets. On June 10 and 11, 20–21 days after the eggs were laid, a new, mated queen from the same supplier was marked, weighed, and added to QMCs containing enough workers following the protocol described above. The evaluation and performance of all subsequent bioassays was staggered within cages (unless stated otherwise) according to when the queen was added to the QMC (day 0). In total, five QMCs were composed with workers maternally exposed to diflubenzuron or control treatment (CTRL-2 and DIF-2), and six and seven QMCs were made with workers maternally exposed to pyriproxyfen and methoxyfenozide respectively (PYR-2 and MET-2). QMCs were given feeders containing untreated sucrose solution, pollen supplement, and water and were maintained as previously described in incubator 1.

#### Assessments and sampling

Assessments including egg production, pollen supplement consumption and retinue behavior were performed as described for Round 1. Retinue behavior was assessed four, five, and six days after queens were added to cages (day 0) for all cages (n = 5 cages CTRL-2, n = 5 DIF-2, n = 6 PYR-2, n = 7 MET-2). See S1 Table for details on the placement of cages in incubator 2. Hatching was assessed by sampling plates of eggs from QMCs on June 17, or days 6 or 7 (5 QMCs CTRL-2, 5 DIF-2, 5 MET-2, 6 PYR-2). Hatching was then assessed as described above, (n = 902 eggs CTRL-2, n = 877 DIF-2, n = 1029 PYR-2, n = 1099 MET-2, see S2 Data). On June 18 (days 7 or 8), 10 adult workers were sampled from cages.

### Statistical analysis

Poisson log-linear generalized estimating equations (GEE) with an auto-regressive (AR-1) correlation matrix structure were implemented to assess treatment related changes in egg production and worker retinue response over time. In an AR-1 correlation matrix structure, the correlations between data points within a subject are assumed to decay as they become further apart, and it is therefore the most appropriate for clustered longitudinal data [81]. Of the available distributions [82], Poisson log-linear distribution was selected based on the data’s characteristics [81]. For Round 2, the first two days of egg laying were excluded from the analysis to prevent model singularity. Time (day) was treated as a continuous variable, treatment was categorical, and Wald chi-square post hoc tests were used to determine significance. Pesticides are known to cause changes in behavior over time [45, 83], and therefore, interaction terms were included in GEE models. Post hoc Dunnett’s tests were used to determine significance between each treatment group and the Ctrl group.

A binomial generalized linear mixed effects model (GLMM) was used to assess the effects of maternal treatment on the number of successful adult eclosions with insect container treated as a random effect. Similarly, binomial GLMMs were used to assess hatching rates of eggs collected in Round 1 with QMC (queen) and the day that they were laid treated as random effects. In Round 2, eggs were sampled once per queen, so, to avoid singularity, only plate (queen) was used as a random effect. Post hoc Dunnett’s tests were again used to evaluate significance between treatment groups and the Ctrl group for both GLMM analyses. To avoid violating assumptions of normality, Kruskal-Wallis one-way analysis of variance with post-hoc Dunn’s tests were used to assess differences in the weight change of queens during QMC experiments, the weight of eclosing adult bees by treatment, pollen consumption, the total sum of eggs laid by queens, the number of larvae eclosed from eggs with deformed cuticle, and the timing of adult eclosion by treatment group. Kaplan Meier survivorship analysis was used to evaluate treatment dependent differences in the survival of adult and queen bees. All statistical analyses were performed using R version 4.0.4 (Boston, MA, USA). Differences in ovary masses were evaluated using a one-way ANOVA after confirming equal variance (Levene test, p = 0.51) and normality (Shapiro test, p > 0.19 for all groups).

Statistical analyses of the mass spectrometry data were performed within Perseus using student’s T tests to compare protein expression between each treatment group and the control. This is a typical approach in proteomics, where it is not feasible to test every protein for normality and equal variance, dictating different tests to be used. Instead, all proteins are subjected to the same test and global false discovery rates (FDRs) are controlled (in this case, using the Benjamini-Hochberg correction to 5% FDR).

## Results

### Round 1: Putative queen exposure via workers

#### Bioassay results

**Fig 4:**
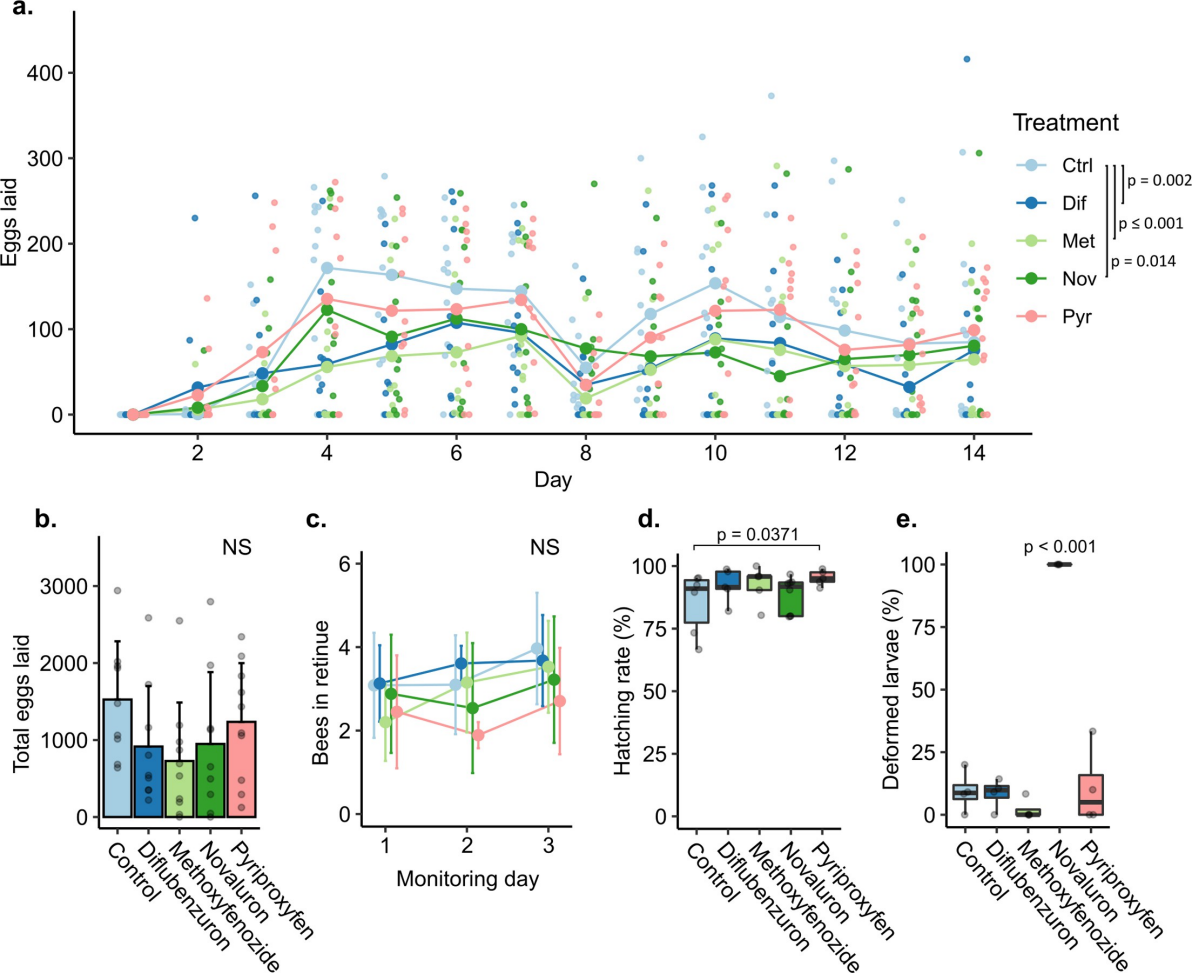
Queen and worker exposure bioassay results. Error bars represent standard deviations. Boxes represent the interquartile range, bars indicate the median, and whiskers span 1.5 times the interquartile range. NS = not significant. Unless otherwise stated, each dot represents a biological replicate. a) Eggs laid by queens in each exposure group were counted daily. Lines and large dots represent average eggs laid across replicates (queens). Small dots depict eggs laid by individual queens. b) Total eggs laid over the course of the experiment (14 d). c) number of bees participating in queen retinues. d) Egg hatching rates. e) Frequency of deformed cuticle phenotype in newly hatched larvae. *** p < 0.001.

The worker diets containing diflubenzuron, methoxyfenozide, and the positive control novaluron had a significant negative effect on daily egg laying relative to the control diet (GEE, n = 10 cages, DIF-1: β = -0.407, Dunnett’s test p = 0.002; MET-1: β = -0.934, p≤0.001; POS-CTRL: β = -0.341, p = 0.014) (Fig 4A). None of the treatments had a significant effect on the total number of eggs laid (Kruskal-Wallis, n = 9–10 cages, chi squared = 6.21, p = 0.184) (Fig 4B). Treated worker diets had no effect on retinue response in Round 1 (GEE, n = 6 cages, all groups Dunnett’s tests p˃0.05 (Fig 4C). See S3 Table for a summary of the results of the GEE analyses. Pyriproxyfen had a significant positive effect on hatching rate relative to Ctrl (GLMM, n = 330–682 eggs, PYR-1: Z = 2.56, Dunnett’s test p = 0.0371, and novaluron treatment resulted in significantly more first instar larvae eclosing with deformed cuticles relative to all treatment groups (Kruskal-Wallis, chi-squared = 170, p≤0.001, Dunn’s test CTRL-1: POS-CTRL, MET-1: POS-CTRL, DIF: 1- POS-CTRL, PYR-1: POS-CTRL: p≤0.001) (Fig 4D and 4E).

No differences were detected between treatment groups in the consumption of pollen supplement (Kruskal-Wallis, n = 9–10 cages, chi-squared = 2.00, p = 0.700, and only a single CTRL queen died during the experiment (S1A-S1C Fig). Treatment had no effect on worker survival (Kaplan Meier, chi-squared = 3.1, p = 0.5), and over the two week monitoring period, mortality rates remained below 3.2% on average across all groups. Total numbers of eggs and larvae evaluated for rate of hatching and deformities are in S1D and S1E Fig. No difference was detected between treatment groups in queen weight change (n = 9–10 queens, chi-squared = 3.00, p = 0.600) (S1F Fig). See S1 Data for more details.

#### Proteomics results

We used label-free quantitative proteomics to investigate patterns of protein expression in the ovaries of exposed and unexposed queens (n = 9 control queens, 9 pyriproxyfen, 10 novaluron, 10 methoxyfenozide, and 10 diflubenzuron). We identified 2,816 protein groups in total, 1,451 of which were considered quantified after filtering (see Methods). Since vitellogenin uptake (such as what occurs in the ovaries) and major royal jelly protein (MRJP) expression are among the processes that could be regulated by growth hormones [84–86], we first tested the hypothesis that IGR exposure could cause changes in vitellogenin or major royal jelly protein abundance in the ovaries. MRJP-1, MRJP-3, vitellogenin, and vitellogenin precursor proteins were among those quantified, but their abundances were not different with respect to the control queens (S2 Fig).

**Fig 5:**
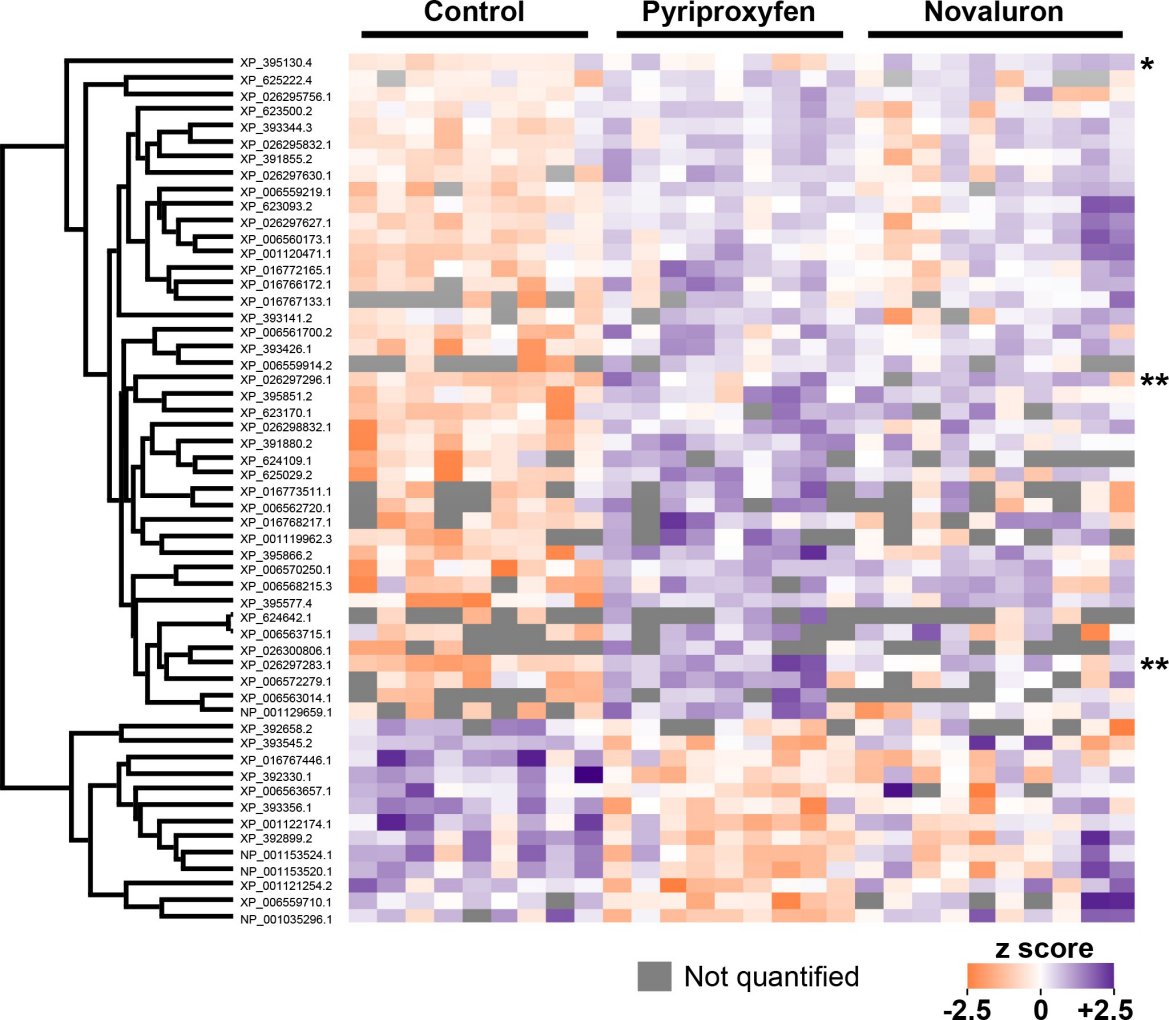
Heat-map of proteins differentially expressed in ovaries of control and pyriproxyfen-exposed queens and between control and novaluron-exposed queens. Protein accessions (NCBI) are shown in the dendrogram. No other proteins were differentially expressed relative to controls (student’s t-tests relative to controls, 5% false discovery rate, Benjamini-Hochberg correction). Hierarchical clustering was performed using Euclidian distance (300 clusters, max 10 iterations). *protein was differentially expressed only in novaluron exposures. **protein was differentially expressed in both novaluron and pyriproxyfen exposures. No asterisk: protein was differentially expressed in pyriproxyfen exposures only.

**Fig 6:**
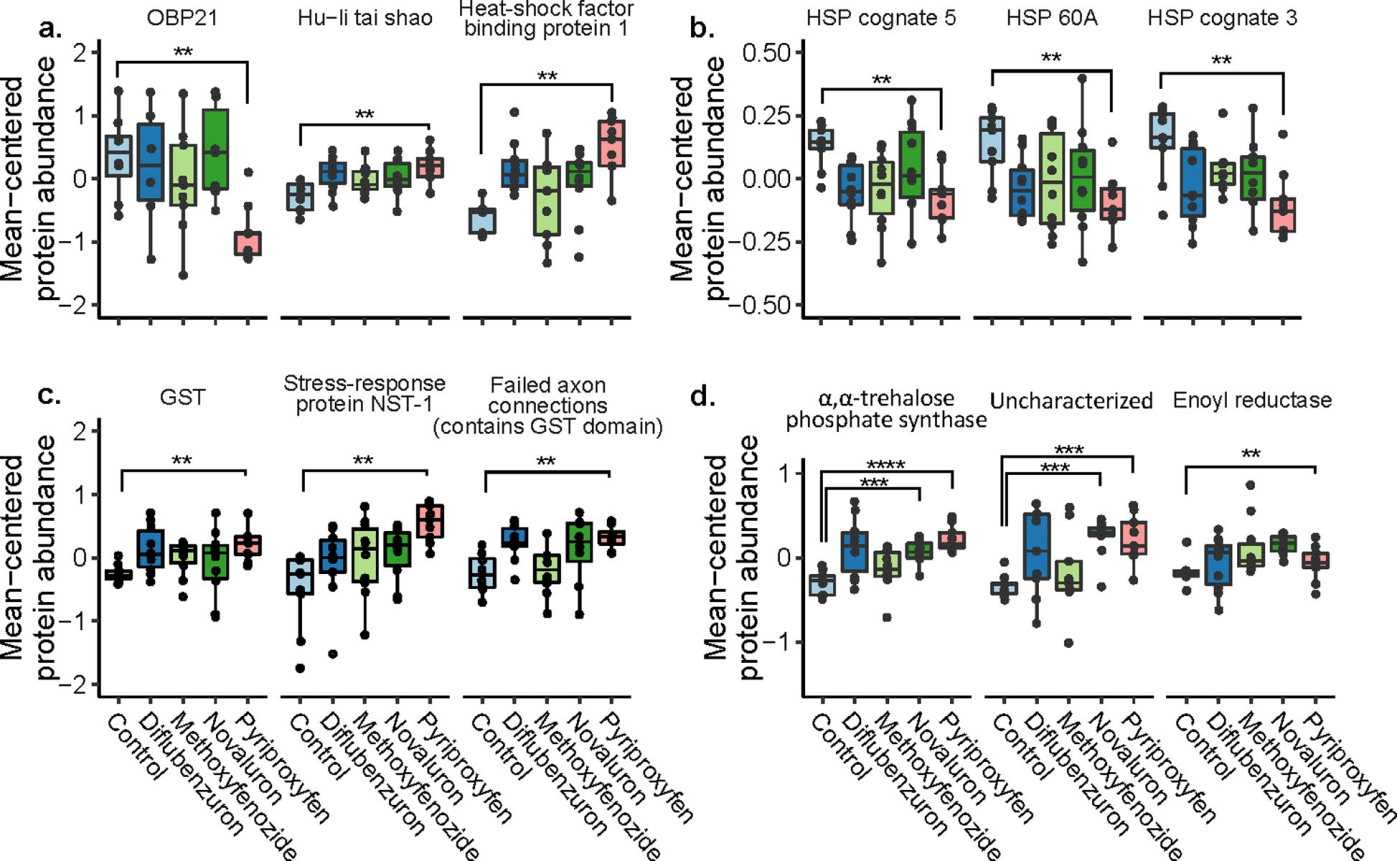
Differentially expressed proteins of interest. Boxes represent the interquartile range, bars indicate the median, and whiskers span 1.5 times the interquartile range. a) OBP21 (NP_001035296.1), Hu-li tai shao protein (XP_016772165.1), and HSF binding protein 1 (NP_001129659.1) expression. b) HSP cognate 5 (NP_001153520.1), HSP 60A (XP_392899.2), and HSP cognate 3 (NP_001153524.1) were downregulated in queens from pyriproxyfen treatments. c) Putative stress-response proteins, GST (glutathione-S-transferase; XP_026295756.1), stress-response protein NST-1 (XP_625029.2), and a GST domain-containing protein (XP_393141.2; officially named “failed axon connections isoform X1”) were upregulated. d) The three proteins differentially expressed in ovaries of novaluron-exposed queens are α,α-trehalose phosphate synthase (XP_026297283.1), uncharacterized protein DDB_G0283697 (XP_026297296.1), and enoyl reductase (XP_395130.4). * p < 0.005, ** p < 0.001, *** p < 0.0001, **** p < 0.00001.

Next, we investigated global patterns of differential protein abundance between exposure groups and found that 55 proteins were significantly different from the control (5% FDR, Benjamini-Hochberg correction; Fig 5). These differences were driven entirely by the pyriproxyfen treatment (54 significantly different proteins) and the novaluron treatment (three significantly different proteins, two of which were also different in the pyriproxyfen group); therefore, these are the only groups displayed. For complete protein quantification data for all groups, see S3 Data. No proteins in the diflubenzuron or methoxyfenozide groups were significantly altered. Proteins of particular interest are shown in Fig 6.

### Round 2: Monitoring workers following putative maternal exposure

**Fig 7:**
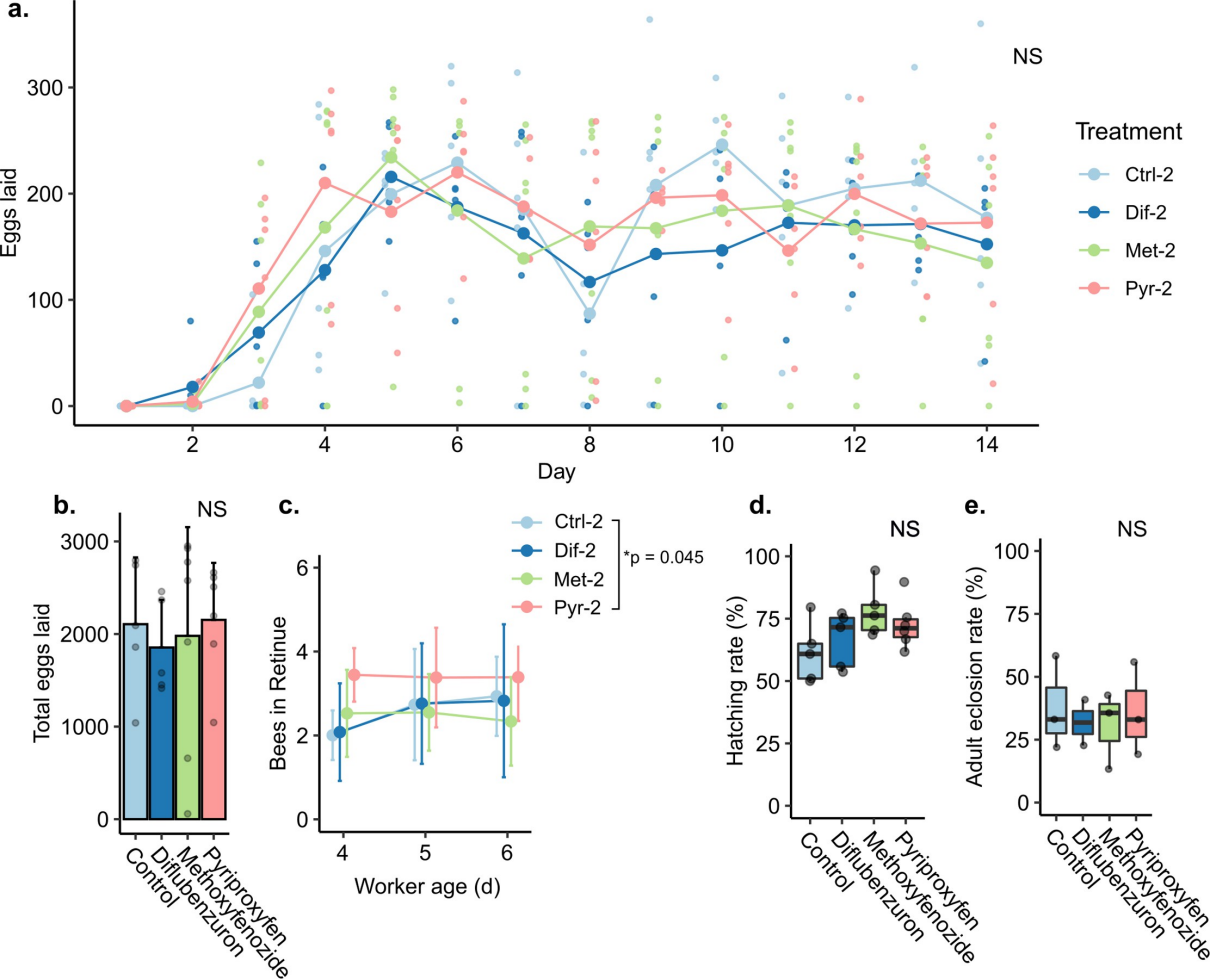
Second generation bioassay results. Error bars represent the standard deviation. Boxes represent the interquartile range, bars indicate the median, and whiskers span 1.5 times the interquartile range. NS = not significant. a & b) eggs laid were recorded daily. c) Number of bees participating in queen retinues. d) Egg hatching rates. e) Adult eclosion rates.

Receiving care from maternally-exposed workers did not have an effect on the laying rates of new queens (GEE, all groups Dunnett’s tests p˃0.05, S4 Table) or their total eggs produced (Fig 7A and 7B, Kruskal-Wallis, n = 5–7 cages, chi squared = 1.34, p = 0.720). Maternal exposure to pyriproxyfen significantly increased worker retinue responsiveness to the new queens relative to control (GEE, n = 5–7 cages, β = 0.719, Dunnett’s test p = 0.045) (Fig 7C). See S4 Table for a summary of the GEE analyses. Receiving care from maternally-exposed workers did not affect the egg hatching rate of eggs laid by new queens (GLMM, n = 877–1099 hatching events, all groups Dunnett’s tests p˃0.05) (Fig 7D) or rate of adult eclosions relative to controls (GLMM, n = 508–1035 adult eclosion events, all groups Dunnett’s tests p˃0.05) (Fig 7E).

**Fig 8:**
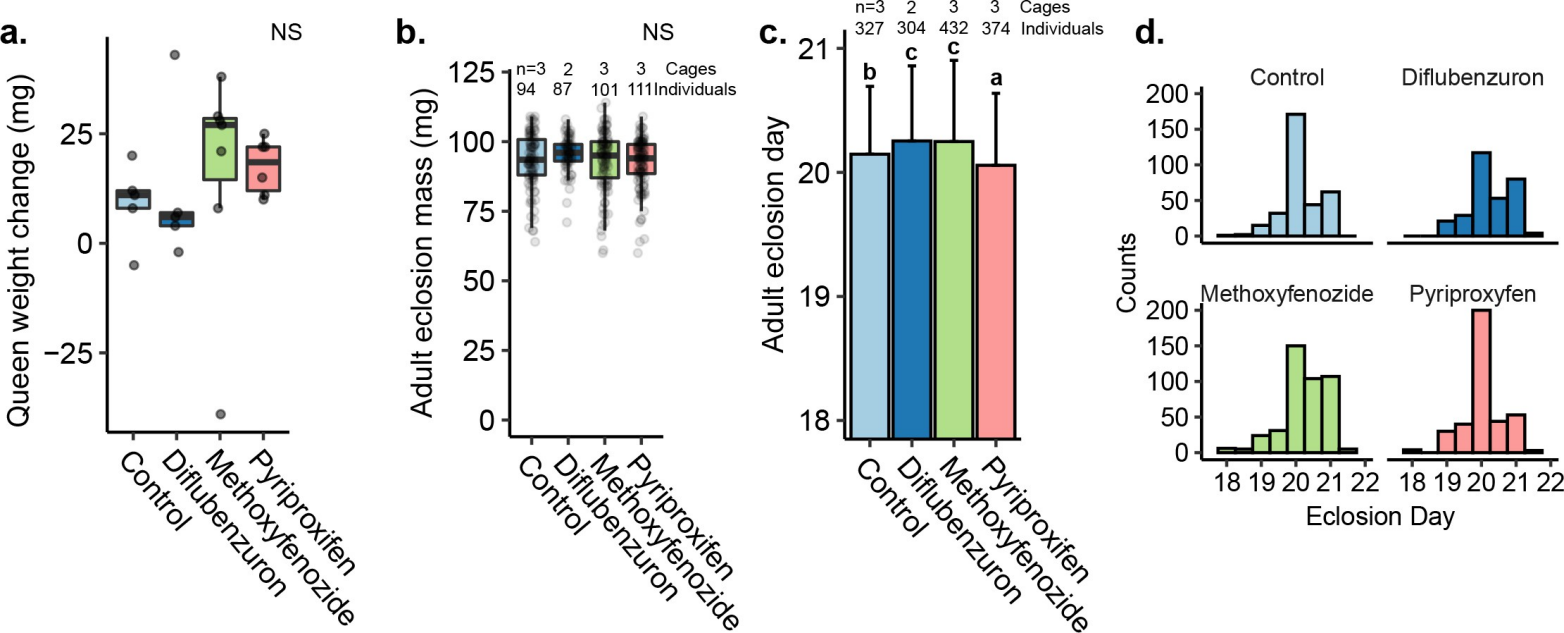
Queen weight change, adult eclosion masses, and timing of adult eclosion for Round 2 cage-trials. Boxes represent the interquartile range, bars indicate the median, and whiskers span 1.5 times the interquartile range. NS = not significant. a) Adult queen weight change during the experiment. Each dot represents one queen. b) Adult worker eclosion masses. Each dot represents one worker (technical replicate). Biological replicates (cages) are shown above each box. c) Average adult eclosion day. Error bars indicate standard deviation and letters indicate statistically significant differences. d) Total individuals eclosing on each day.

Treatment also had no effect on worker pollen consumption (Kruskal-Wallis, n = 5–7 cages, chi-squared = 2.00, p = 0.700), queen weight change (n = 5–7, chi-squared = 3.00, p = 0.600), or weight at adult eclosion (n = 69–111 bees, chi-squared = 4.95, p = 0.176) (Figs S3 and 8A and 8B). However, treatment had a significant effect on the timing of adult eclosion. Maternal exposure to diflubenzuron and methoxyfenozide resulted in significantly longer average time to adult eclosion relative to maternal exposure to pyriproxyfen or the control group (Kuskal-Wallis, n = 304–432 bees, chi-squared = 35.705, p≤0.001, Dunn’s test CTRL-DIF: Z = -2.415, p = 0.0236; CTRL-MET: Z = -3.272, p = 0.00214; DIF-MET: Z = -0.634, p = 0.526; CTRL-PYR: Z = 4.356, p≤0.001; MET-PYR: Z = 5.434, p≤0.001) (Fig 8C and 8D). Maternal pesticide treatment had no effect on worker survival (Kaplan Meier, n = 281–417 bees, chi-squared = 1.6, p = 0.700). Over the two week monitoring period, mortality rates remained below 1.7% on average across all groups, and no queen death was observed. See S2 Data for more details.

## Discussion

In this work, we demonstrated that indirectly exposing queens to a juvenile hormone (JH) analog (pyriproxyfen) through the workers’ diet increased egg hatching rates, and offspring of queens cared for by exposed workers showed an increased responsiveness to novel queens. In contrast, the same doses of the ecdysteroid agonist, methoxyfenozide, and the chitin synthesis inhibitor diflubenzuron, which negatively affected daily oviposition rates, did not affect the behavior of offspring. Based on these results and previously reported findings [59], we hypothesized that there may be transovarial effects of IGD pesticides, and sought to investigate this by conducting shot-gun proteomics on ovary tissue. Interestingly, we found that pyriproxyfen exposure had the most dramatic effect on ovary protein expression, and some of the differentially expressed proteins have well-defined stress response functions, including several heat-shock proteins. By contrast, the positive control, novaluron (administered at a dose 20 times higher than the other treatments), which negatively affected queen oviposition and larval deformity rates, caused only a small change in protein expression with just three differentially expressed proteins (two overlapping with the pyriproxyfen group). Without mechanistic studies, we cannot definitively say which, if any, of the differentially expressed proteins may have led to increased hatching rates in the pyriproxyfen group, but an intriguing hypothesis to test will be the relationship between maternal heat-shock protein expression and egg hatching rates.

While this work did not determine whether the observed effects were due to direct exposure of queens to the pesticide treatments through worker trophallactic feedings or due to effects of the treatments on the worker caretakers, the former route is supported by the absence of any observed behavioral effects on workers directly exposed to the treatments, particularly for pyriproxyfen. In workers, rising JH titers accelerate the behavioral transition away from in-hive task like queen care [87, 88]. If pyriproxyfen had affected workers, the anticipated effect would have been decreased queen care behaviors and decreased queen oviposition, but this was not observed. Whether queens were exposed directly or indirectly, the effects we observed are important because pyriproxyfen and other insect growth regulating pesticides are generally not thought to affect honey bee queen offspring following an exposure event. However, here we see that adult exposure can affect honey bee reproduction by affecting queen ovarian tissue, embryo survival, and the behavior of worker offspring.

In insects, maternal environment is known to affect various biological characteristics in offspring including survival, developmental timing, onset of diapause, egg production, size, and pupal and adult weight [89–93]. Likewise, honey bees have evolved different responses to stress and natural conditions that affect aspects of reproduction. In temperate climates [94], and perhaps to some extent in warmer regions as well [95], the queen will cease laying or greatly reduce her output of eggs during winter months when temperatures are generally cooler and there is a dearth of resources available to foragers. Similarly, prior to swarming, queens will lose weight presumedly due to decreased worker provisioning [96]. Recently, colony nutrient deprivation has been shown to affect the size and survivorship of honey bee queen-laid embryos [97]. Curiously, queens in pollen restricted colonies laid larger eggs that survive to adult eclosion in foster colonies at higher rates than eggs from unrestricted colonies. This suggests that, like other insect taxa, maternal stress can affect honey bee worker development, in this case by increasing maternal investment in her offspring. This type of compensatory response may be particularly advantageous for social insects whose long-term survival is heavily dependent on the continued production of new worker bees [67]. Indeed, this type of response has also been observed in starved worker larvae who demonstrate increased responsiveness to queens as adults [28]. Worker care is critical to a queen’s success; without workers to care for her, the queen will cease to produce eggs [20, 67], and relatedly, workers may be less interested in caring for a poorly performing queen [34, 98]. This equation must be precisely balanced, or colony populations dwindle, which may eventually lead to colony loss [58, 99]. The work described here, which demonstrated increased responsiveness to queens in the worker offspring of queens exposed to pyriproxyfen, may describe another compensatory response to stress on the part of queen honey bees.

In colony studies, high doses of JH analogs have been shown to be reproductive toxins, resulting in brood and embryo mortality following colony-level feedings [53, 100–102]. Our proteomics analysis of queen ovaries shows that Glutathione S-transferase S1, a putative detoxification enzyme [103], was upregulated following pyriproxyfen exposure, suggesting that some pesticide related stress was occurring in ovarian tissues. Another protein, named Stress response protein NST1, was also upregulated in ovaries from queens exposed to pyriproxyfen; however, exactly why this is called a “stress response protein” is unclear. The protein has GO terms linked to nervous system development (GO:0007399), gamete generation (GO:0007276), movement of cells during oogenesis (GO:0007298), and germ cell migration (GO:0008354), likely driven by microtubule polymerization (GO:00311100). Moreover, the broader pattern of protein expression is not entirely consistent with the hypothesis that pyriproxyfen triggers a canonical stress response.

Among the other differentially expressed proteins were Hu-li tai shao protein, which, like Stress response protein NST1, was upregulated in pyriproxyfen-treated queens, and has been previously linked to oogenesis in *Drosophila melanogaster* [104]. OBP21, which was downregulated in pyriproxyfen exposed queens, is a putative soluble odorant receptor [105]; however, OBPs have been repeatedly identified in non-olfactory tissues, and are abundant in honey bee spermathecal fluid and drone ejaculates [106, 107]. We postulate that they could have dual roles in hormone trafficking or signaling. Heat-shock factor binding protein (HSFBP)1, which was upregulated, acts as a negative regulator of the heat-shock response in other animals [108], and indeed, patterns of heat-shock protein (HSP) expression here were correspondingly downregulated.

HSPs are stress response proteins linked to extreme temperatures [75, 107, 109–113], pesticide exposure [114, 115], and viral infections [116] in honey bees, however, the expression patterns observed in pyriproxyfen exposed queen tissue are inconsistent with a typical stress response. While previous work cited above has shown that HSPs are upregulated in response to diverse stressors, here, the three HSPs identified were downregulated. This suggests that the observed behavioral shift in queen offspring and the increased hatching rates following maternal pyriproxyfen exposure may not be solely attributable to stress.

In solitary insects, JH induces vitellogenesis related to egg production [117], but in worker honey bees, its role has shifted. Rising JH levels in the hemolymph of worker bees is associated with behavioral maturation and triggers a shift from nursing and other in-hive activities to foraging [87, 118, 119]. Here, worker exposure to a JH analog did not result in reduced queen care behaviors, suggesting it had minimal impacts on worker behavioral maturation at the dose tested. It did, however, affect reproduction in a seemingly positive manner, and pyriproxyfen was the only maternal treatment that did not result in delayed adult eclosion relative to control.

During development, JH regulates ovarian development [120, 121], and supplementing queen larval diet with JH has been shown to increase the reproductive quality of queens [122]. While increasing JH titers are not known to play a role in oogenesis in adult workers, and in fact, may have a slight inhibitory effect [123], in adult queens, its function has not been well explored. Previous work has shown that hemolymph titers of JH are higher in pre-vitellogenic queens or queens prevented from laying, suggesting that JH may inhibit oogenesis in honey bee queens [124]. However, the referenced work did not introduce exogenous JH to queens but measured the naturally occurring levels in queens that either could not lay or were not likely to lay given their reproductive status. In this work, we indirectly exposed queens to JH through worker diet but did not measure JH titers in the hemolymph. Mated queens in QMCs were given sufficient space to lay, and we observed no differences in the oviposition rates of pyriproxyfen exposed queens.

Beyond the curious effects of pyriproxyfen, the results described here are largely consistent with previous work, with a few novel observations. As shown in previous studies with QMCs [49, 59, 66], egg production rates generally increased over the two week monitoring periods, but apparent declines midway through the experiments may be attributed to disruptive sampling procedures, suggesting that such activities should be limited. Like oviposition rates, retinue responses of workers generally increased over time. These observations may be attributable to the bees adapting to the cage environment, and their consistency with previous experiments demonstrating realistic responses of bees to stimuli in QMCs [49, 66] suggests that the novel conditions introduced here such as the red light in the incubators was not overly disruptive to the bees. The spectrum of the light produced by the red lights used in this work was not described and may have contained shorter wavelength light (below 650 nm) which would be visible to bees [125].

Previous work has shown that ten-fold higher doses of diflubenzuron and methoxyfenozide cause decreased hatching rates without affecting the total number of eggs produced by exposed queens [59]. Here, hatching rates were not affected at a lower dose, and methoxyfenozide, diflubenzuron, and novaluron caused decreased daily oviposition rates. This is not inconsistent with previous findings, given that the referenced study considered the total sum of eggs laid during the treatment period and not daily oviposition rates (here, we tested both the summed total and daily oviposition rates, and the latter was more sensitive at detecting differences). However, twice the dose of novaluron compared to what was used in the previous study did not change hatching rates whereas in the previous study, fewer eggs from novaluron exposed queens had hatched three days after they were laid. However, we did observe that the majority of larvae hatching from novaluron exposed eggs were severely deformed and not likely to be viable. It is notable that in this work, egg hatching was observed for an extra day. While the three day temporal window was selected based on the reported variation in time to first instar eclosion for worker honey bees [126], it is possible that following maternal exposure to reproductively toxic pesticides, damaged eggs may initiate hatching later than healthy, less affected eggs. Therefore, when conducting embryo-fetal development studies on honey bees, we recommend examining hatching rates over four days to improve sensitivity of these assays.

Despite the obvious physiological effects of novaluron on embryos, only three proteins were significantly different in novaluron-treated queen ovarian tissue (Fig 7D). The role these three proteins may play in oogenesis or embryogenesis is unclear, and it will be particularly important to elucidate the roles of the two proteins differentially expressed in both novaluron and pyriproxyfen treatments. Enoyl-ACP reductase (XP_395130.4) was upregulated only in the novaluron group, and this protein is thought to be involved in fatty acid metabolism and synthesis of cuticular hydrocarbons [127]. Of the proteins differentially expressed in both novaluron and pyriproxyfen groups, one is currently uncharacterized (XP_026297296.1), and the other is annotated as α,α-trehalose phosphate synthase (XP_026297283.1). Trehalose phosphate synthase is a key enzyme in the trehalose biosynthetic pathway [128, 129]. Trehalose is an important hemolymph sugar and has been linked to tolerance of desiccation stress [130] and temperature stress [131, 132] in insects. Interestingly, insects unable to hydrolyze trehalose have abnormal phenotypes, such as weight loss, lethal metamorphosis, disrupted chitin synthesis, and poor stress recovery, among others, and trehalose hydrolase expression is thought to be under hormonal control [129]. Trehalose is considered to be an anti-stress metabolite, providing a source of energy while stabilizing proteins and membranes after exposure to extreme temperatures.

Further examination of the proteins affected by novaluron or pyriproxyfen exposure will elucidate their roles in oogenesis and embryogenesis, but the limited effects on the maternal ovarian proteome caused by novaluron exposure suggest that the effects on the hatching first instar larvae may have been due to contact via workers or maternal offloading of novaluron to developing eggs, though the former may be unlikely as the eggs were removed within a day of being laid. Previous work had shown that IGDs can persist in insect eggs following maternal exposure, resulting in embryo mortality [133]. During honey bee embryogenesis, chitin synthesis does not begin until after eggs are laid [134], therefore if novaluron were offloaded into the oocytes, major effects might not become apparent until after the eggs were laid and chitin synthesis had begun.

The methods used in this work allow for precision monitoring of queens and their reproductive output, which may eventually lead to insights regarding the physiology of queens and the long-term impacts of stress and disrupted hormone homeostasis on colonies. Here, we identified vertical effects of pyriproxyfen, a JH analog used in honey bee-pollinated crops during and adjacent to the blooming period. The doses of pyriproxyfen, diflubenzuron, and methoxyfenozide used are similar to residues of novaluron in freshly collected pollen [63], which bees have been shown to prefer [135], therefore we theorize that this dose may represent a worst-case scenario for honey bee colonies. Regardless, the finding that maternal agrochemical exposure can behaviorally alter the next cohort of worker bees is significant. Already, it is well known that perturbations to a colony’s population balance can affect the longevity of the colony unit [58, 83, 99], but little is known about how stressors on queens can influence colony dynamics. This work suggests that while queens may not be wholly protected against the effects of external stress, they may have evolved strategies to compensate for such effects. Future work exploring the effects on worker offspring physiology and the longitudinal consequences for pyriproxyfen exposed colonies may help elucidate the consequences of maternal pyriproxyfen exposure in honey bees.

## Supporting information

S1 Fig First generation pollen consumption, queen weight change, and worker death. Error bars represent standard deviation. Boxes represent the interquartile range, bars indicate the median, and whiskers span 1.5 times the interquartile range. Each dot represents a different replicate. No significant differences were found in any comparisons. a) Total pollen consumption over the course of the experiment. b) Queen weight change 14 d after initial worker-mediated exposure. c) Number of workers dying over the course of the experiment. d) Total number of eggs evaluated to determine hatching rates. e) Total number of larvae imaged for evaluation of cuticle deformities. f) Ovary masses of exposed queens.
(TIF)

S2 Fig Expression of major royal jelly proteins and vitellogenin proteins in ovaries of queens. LFQ = label-free quantitation. NS = not significant (Benjamini-Hochberg correction, 5% FDR). Boxes represent the interquartile range, bars indicate the median, and whiskers span 1.5 times the interquartile range.
(TIF)

S3 Fig Second generation pollen consumption. No significant differences were found. Boxes represent the interquartile range, bars indicate the median, and whiskers span 1.5 times the interquartile range.
(TIF)

S1 Table The identity of each QMC placed in incubator 2. Retinue monitoring spaces on shelves 1–4 for the first generation (Round 1) queen and worker exposure and the second worker generation (Round 2) after maternal exposure. Space numbers correspond to numbered spaces depicted in Fig 2.
(DOC)

S2 Table The QMC source identity of plates placed in foster colonies, the identity of the insect container the plates were placed in once the eggs had been reared to pupation, the number of eggs present on each plate, and the number of adults eclosing in each container. (DOC)

S3 Table Summary of the results of the GEE analyses for daily egg laying and worker retinue responses–Round 1. (DOC)

S4 Table Summary of the results of the GEE analyses for daily egg laying and worker retinue responses–Round 2. (DOC)

S1 Data Round 1 data including pollen consumption, queen weight change, retinue observations, egg production, worker mortality, egg eclosion rates, and rates of larval deformities. (XLSX)

S2 Data Round 2 data including pollen consumption, queen weight change, retinue observations, egg production, worker mortality, egg eclosion rates, and adult emergence timing and mass. (XLSX)

S3 Data Mass spectrometry sample metadata and ovary protein quantification data. (XLSX)
