# Correction: Indirect exposure to insect growth disruptors affects honey bee (*Apis mellifera*) reproductive behaviors and ovarian protein expression

**DOI:** 10.1371/journal.pone.0330642

**Published:** 2025-08-20

**Authors:** The PLOS One Staff

In Fig 1, the arrows that depicts temporal directionality are missing. Please see the correct Fig 1 here.

**Fig 1:**
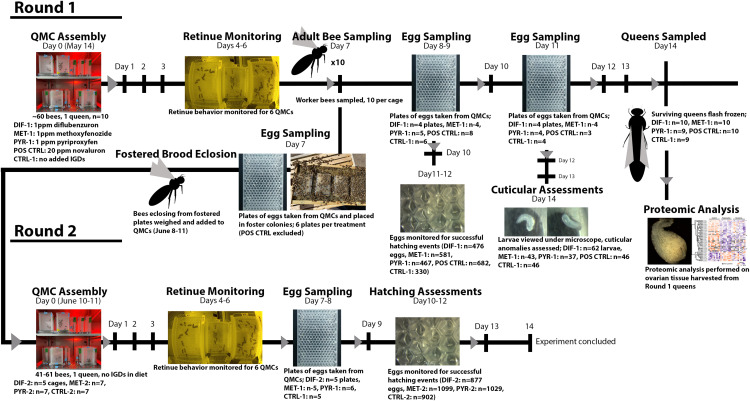
Graphical representation of experimental timeline.

The publisher apologizes for the error.
