# Supplementary figures and images for: Indirect exposure to insect growth disruptors affects honey bee (Apis mellifera) reproductive behaviors and ovarian protein expression
Source: PLoS One. 2023 Oct 2;18(10):e0292176. doi: 10.1371/journal.pone.0292176 (PMC10545116; doi:10.1371/journal.pone.0292176)

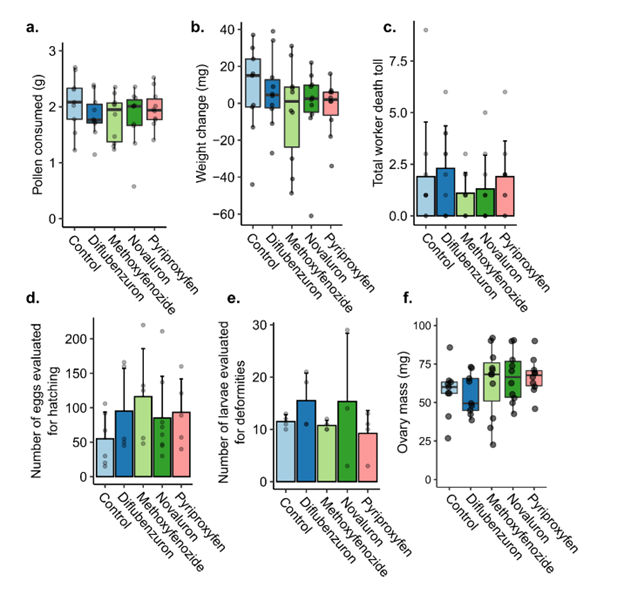

Supplement: S1 Fig — Error bars represent standard deviation. Boxes represent the interquartile range, bars indicate the median, and whiskers span 1.5 times the interquartile range. Each dot represents a different replicate. No significant differences were found in any comparisons. a) Total pollen consumption over the course of the experiment. b) Queen weight change 14 d after initial worker-mediated exposure. c) Number of workers dying over the course of the experiment. d) Total number of eggs evaluated to determine hatching rates. e) Total number of larvae imaged for evaluation of cuticle deformities. f) Ovary masses of exposed queens. (TIF) [file pone.0292176.s001.tif]

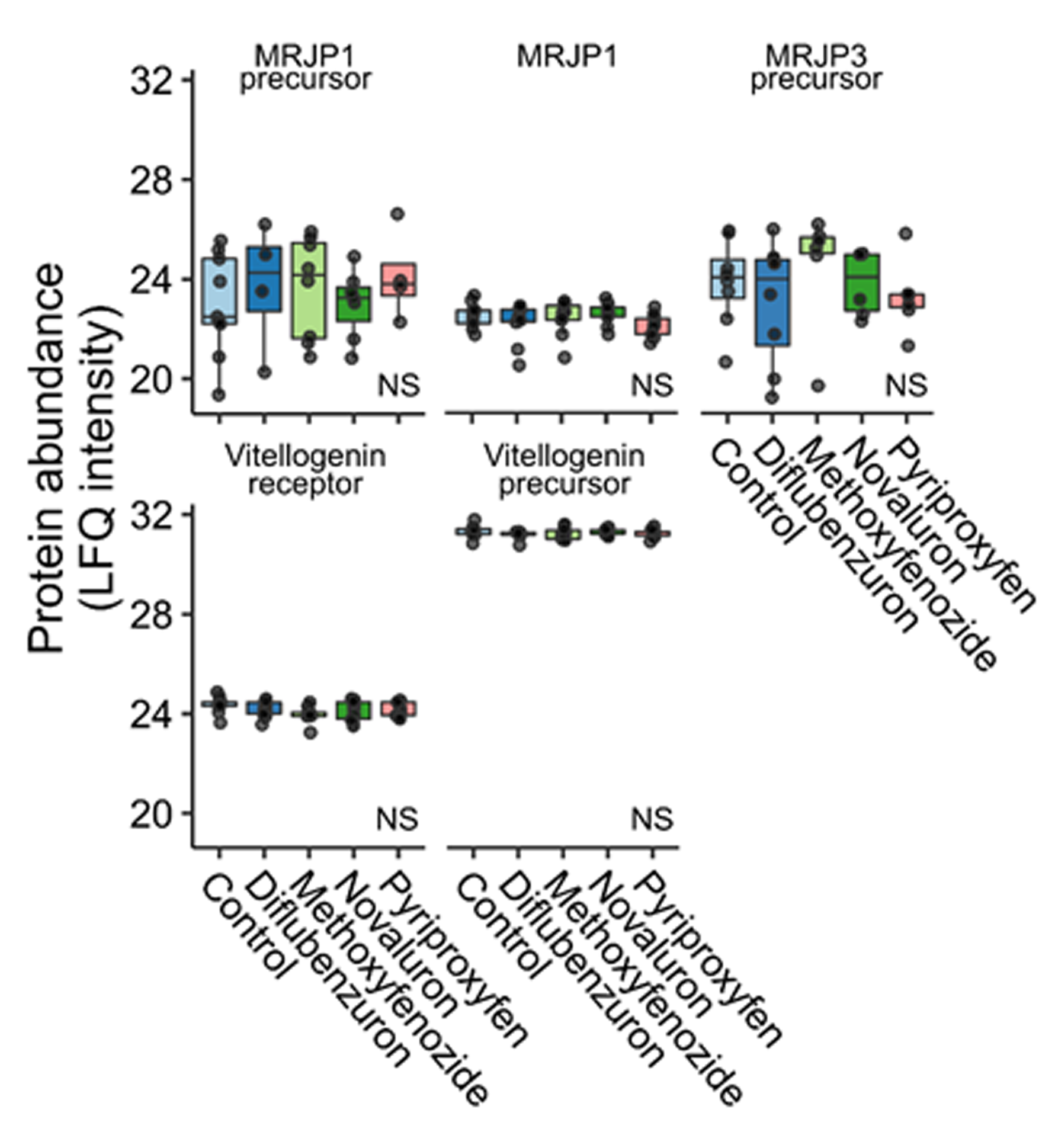

Supplement: S2 Fig — LFQ = label-free quantitation. NS = not significant (Benjamini-Hochberg correction, 5% FDR). Boxes represent the interquartile range, bars indicate the median, and whiskers span 1.5 times the interquartile range. (TIF) [file pone.0292176.s002.tif]

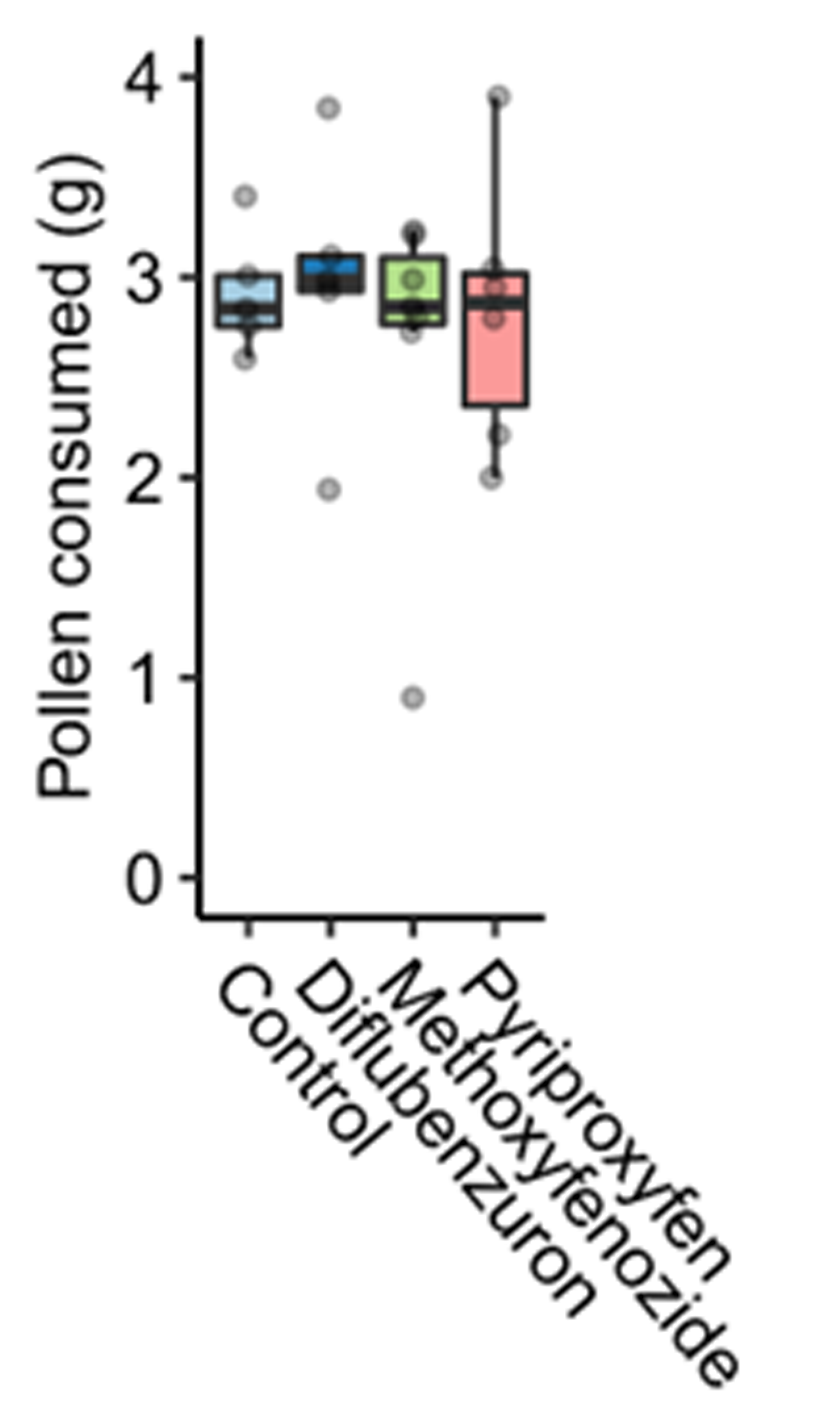

Supplement: S3 Fig — No significant differences were found. Boxes represent the interquartile range, bars indicate the median, and whiskers span 1.5 times the interquartile range. (TIF) [file pone.0292176.s003.tif]
