# Supplementary material for: Indirect exposure to insect growth disruptors affects honey bee (Apis mellifera) reproductive behaviors and ovarian protein expression
Source: PLoS One. 2023 Oct 2;18(10):e0292176. doi: 10.1371/journal.pone.0292176 (PMC10545116; doi:10.1371/journal.pone.0292176)
Supplement: S1 Table — Retinue monitoring spaces on shelves 1–4 for the first generation (Round 1) queen and worker exposure and the second worker generation (Round 2) after maternal exposure. Space numbers correspond to numbered spaces depicted in Fig 2. (DOC) [file pone.0292176.s004.doc]

| **Round 1: Putative Queen Exposure Via Workers** | | |  |
| --- | --- | --- | --- |
|  |
| ***Shelf 1*** | | |  |
| 1. CTRL 1 | 2. NOV 1 | 3. DIF 1 |  |
| 4. MET 1 | 5. PYR 1 | 6. CTRL 2 |  |
| 7. NOV 2 | 8. DIF 2 | 9. MET 2 |  |
| ***Shelf 2*** | | |  |
| 1. PYR 2 | 2. CTRL 3 | 3. NOV 3 |  |
| 4. DIF 3 | 5. MET 3 | 6. PYR 3 |  |
| 7. CTRL 4 | 8. NOV 4 | 9. DIF 4 |  |
| ***Shelf 3*** | | |  |
| 1. MET 4 | 2. PYR 4 | 3. CTRL 5 |  |
| 4. NOV 5 | 5. DIF 5 | 6. MET 5 |  |
| 7. PYR 5 | 8. CTRL 6 | 9. NOV 6 |  |
| ***Shelf 4*** | | |  |
| 1. DIF 6 | 2. MET 6 | 3. PYR 6 |  |
| **Round 2: Monitoring Workers Following Putative Maternal Exposure** | | |  |
|  |
| ***Shelf 1*** | | |  |
| CTRL-2 1 | DIF-2 1 | MET-2 1 |  |
| PYR-2 1 | CTRL-2 2 | DIF-2 2 |  |
| ***Shelf 2*** | | |  |
| MET-2 2 | PYR-2 2 | CTRL-2 3 |  |
| DIF-2 4 | CTRL-2 5 | DIF-2 5 |  |
| ***Shelf 3*** | | |  |
| DIF-2 3 | MET-2 3 | PYR-2 3 |  |
| MET-2 5 | MET-2 6 | PYR-2 6 |  |
| ***Shelf 4*** | | |  |
| CTRL-2 4 | MET-2 4 | PYR-2 4 |  |
| PYR-2 5 |  | MET-2 7 |  |
