# Supplementary material for: Indirect exposure to insect growth disruptors affects honey bee (Apis mellifera) reproductive behaviors and ovarian protein expression
Source: PLoS One. 2023 Oct 2;18(10):e0292176. doi: 10.1371/journal.pone.0292176 (PMC10545116; doi:10.1371/journal.pone.0292176)
Supplement: S2 Table — (DOC) [file pone.0292176.s005.doc]

| **QMC** | **Live Insect Container** | **Number of Eggs** | **Adult Eclosions** |
| --- | --- | --- | --- |
| **CTRL 1** | 1 | 108 | 24 |
| **CTRL 4** | 2 | 208 | 182 |
| **CTRL 8** |  | 245 |  |
| **CTRL 6** | 3 | 133 | 121 |
| **CTRL 9** |  | 211 |  |
| **PYR 1** | 4 | 197 | 34 |
| **PYR 2** | 5 | 212 | 146 |
| **PYR 3** |  | 229 |  |
| **PYR 8** | 6 | 195 | 194 |
| **PYR 10** |  | 202 |  |
| **MET 1** | 7 | 203 | 82 |
| **MET 3** | 8 | 82 | 87 |
| **MET 8** |  | 204 |  |
| **MET 9** | 9 | 218 | 263 |
| **MET 10** |  | 45 |  |
| **DIF 1** | 10 | 75 | 44 |
| **DIF 6** |  | 104 |  |
| **DIF 7** | 11 | 213 | 260 |
| **DIF 10** |  | 116 |  |
