# Supplementary material for: Indirect exposure to insect growth disruptors affects honey bee (Apis mellifera) reproductive behaviors and ovarian protein expression
Source: PLoS One. 2023 Oct 2;18(10):e0292176. doi: 10.1371/journal.pone.0292176 (PMC10545116; doi:10.1371/journal.pone.0292176)
Supplement: S3 Table — (DOC) [file pone.0292176.s006.doc]

| **Model: Daily Egg Laying Rates** |  |  |  |
| --- | --- | --- | --- |
| Predictor | Estimate ± S.E. | Wald chi-square | Dunnett’s test p-value (comparison to Control) |
| Day | 0.0507±0.0203 | 6.23 |  |
| **Diflubenzuron** | **-0.407 ±0.317** | **1.65** | **0.002** |
| **Methoxyfenozide** | **-0.934±0.333** | **7.85** | **≤0.001** |
| **Novaluron** | **-0.341±0.298** | **1.31** | **0.014** |
| Pyriproxyfen | -0.075±0.274 | 0.076 | 0.574 |
| Day: Diflubenzuron | -0.013±0.035 | 0.13 |  |
| Day: Methoxyfenozide | -0.030±0.034 | 0.77 |  |
| Day: Novaluron | -0.008±0.032 | 0.056 |  |
| Day: Pyriproxyfen | -0.009±0.028 | 0.10 |  |
| **Model: Worker Retinue Response** |  |  |  |
| Predictor | Estimate ± S.E. | Wald chi-square | p-value |
| Day | 0.134±0.092 | 2.12 |  |
| Diflubenzuron | 0.173±0.252 | 0.47 | 0.969 |
| Methoxyfenozide | -0.306±0.285 | 1.15 | 0.536 |
| Novaluron | -0.048± 0.369 | 0.02 | 0.717 |
| Pyriproxyfen | -0.146±0.371 | 0.16 | 0.068 |
| Day: Diflubenzuron | -0.072± 0.117 | 0.37 |  |
| Day: Methoxyfenozide | 0.092± 0.124 | 0.55 |  |
| Day: Novaluron | -0.040± 0.166 | 0.06 |  |
| Day: Pyriproxyfen | -0.072±0.170 | 0.18 |  |
