# Supplementary material for: Indirect exposure to insect growth disruptors affects honey bee (Apis mellifera) reproductive behaviors and ovarian protein expression
Source: PLoS One. 2023 Oct 2;18(10):e0292176. doi: 10.1371/journal.pone.0292176 (PMC10545116; doi:10.1371/journal.pone.0292176)
Supplement: S4 Table — (DOC) [file pone.0292176.s007.doc]

| **Model: Daily Egg Laying Rates** |  |  |  |
| --- | --- | --- | --- |
| Predictor | Estimate ± S.E. | Wald chi-square | p-value |
| Day | 0.102±0.021 | 24.42 |  |
| Diflubenzuron | 0.016±0.257 | ≤0.01 | 0.875 |
| Methoxyfenozide | 0.245±0.267 | 0.65 | 0.993 |
| Pyriproxyfen | 0.318±0.303 | 1.10 | 0.954 |
| Day: Diflubenzuron | -0.015±0.023 | 0.45 |  |
| Day: Methoxyfenozide | -0.029±0.023 | 1.61 |  |
| Day: Pyriproxyfen | -0.022±0.028 | 0.60 |  |
| **Model: Worker Retinue Response** |  |  |  |
| Predictor | Estimate ± S.E. | Wald chi-square | p-value |
| Day | 0.282±0.122 | 5.35 |  |
| Diflubenzuron | 0.035±0.299 | 0.01 | 0.990 |
| Methoxyfenozide | 0.234±0.426 | 0.30 | 0.981 |
| **Pyriproxyfen** | **0.719±0.292** | **6.09** | **0.045** |
| Day: Diflubenzuron | -0.035±0.146 | 0.06 |  |
| Day: Methoxyfenozide | -0.131±0.207 | 20.40 |  |
| **Day: Pyriproxyfen** | **-0.216±0.132** | **2.67** |  |
